# The Hospital. Nursing Section

**Published:** 1906-03-03

**Authors:** 


### The Hospital.

**Nursing Section.**

Contributions for "The Hospital," should be addressed to the Editor, "The Hospital" Nursing Section, 28 & 29 Southampton Street, Strand, London, W.C.

No. 1,014.—Vol. XXXIX. SATURDAY, MARCH 3, 1906.

### Notes on News from the Nursing World.

### THE PRINCE OF WALES AT AN INDIAN STATION HOSPITAL.

On the 6th of last month, the Prince of Wales paid an informal visit to the Station Hospital at Bangalore. On the previous day, his Royal Highness and the Princess of Wales had an enthusiastic greeting from the British natives and residents, followed in the evening by a reception at the Residency, to which the members of the nursing staff at the Station Hospital were invited. The next morning, the lady superintendent and the sisters of Queen Alexandra's Imperial Military Nursing Service for India, stood at the top of the stairs leading to the wards to receive the Prince. After they had each been presented to him, they followed him into the wards, which were looking very pretty, with roses in pots and vases on the tables—in fact, roses everywhere. The orderlies were in their places, and they and the patients, who had engaged in many discussions as to the height of the Prince, were at last able to judge from sight! "Well, he's a little 'un, isn't he, Sister, but don't his clothes fit!" was the verdict of one Tommy. The Prince remarked upon the lavish display of roses, which he was told all came from the sisters' garden, and after walking through each ward and into the bath-room—laid out ready for an operation, as it also does duty for a theatre—he looked into the duty-room. The Prince consented that a lovely bouquet of white roses, which was to have been presented to the Princess if she had come, should be sent to her at the Residency, and they were at once despatched covered with wet towels and shaded from the sun by an umbrella. In the afternoon, at the Residency garden party, the Princess, after sending for the nursing sisters, remarked that she was sorry they had been disappointed that she did not go to the hospital, but she did not know that they expected her.

### RAISING THE STANDARD FOR QUEEN'S NURSES.

At the meeting of the Council of Queen Victoria's Jubilee Institute for Nurses, last week, it was decided that the qualification as to hospital training for Queen's nurses should be raised "from two to three years at approved hospitals or infirmaries, to include at least two years in a general hospital or infirmary." We conclude that the third year may be devoted to training in fever or other special work. The elevation of the standard to three years cannot fail to be of advantage both to the Institute, the public, and the nurses. We congratulate Miss Amy Hughes upon this wise new departure which has so quickly followed upon her appointment to the post of general superintendent.

### THE MATRONSHIP OF KING'S COLLEGE HOSPITAL.

At the annual Court of Governors of King's College Hospital, last week, the Committee reported the resignation, owing to ill-health, of Miss Monk as sister matron. Our notice of Miss Monk's career should have shown that she has been ward sister at Charing Cross Hospital, as well as night sister at King's College and Charing Cross Hospitals, during the period when both institutions were under the same management. Many letters expressing regret at her retirement have been received by Miss Monk since our authoritative announcement last week, and we may add that no one feels keener sorrow than Miss Monk herself that she is absolutely compelled, by the state of her health, to relinquish her life's work.

### DEVELOPMENTS OF DISTRICT WORK AT SOUTHPORT.

We learn from the 20th annual report of the Southport and Birkdale District Nursing Society, that during last year the night-nursing has been a much more important item than formerly. The Committee are not, of course, able to allow the regular staff, which consists of six nurses, to undertake this work, but, when necessary, outside help is engaged. The sum expended in this direction in 1905 was £21 12s., as compared with £6 10s. in 1904. There was also an entirely new development in the work of the Society during the year. At the instance of the medical officer of health, the Committee agreed to engage an extra nurse competent to combine the duty of looking after and watching over the infants born in Southport for the first twelve months of their life, with the midwifery work of the organisation, a grant of £50 a year towards her salary being made from the borough funds. The nurse was accordingly selected and joined the staff on January 1 of the present year. It will be interesting to see how the experiment answers.

### YORK HOME FOR NURSES.

During the past year great changes have taken place in the York Home for Nurses. For twenty-eight years the institution has been under the management of the Sisterhood of the Holy Cross, but last year the Sisters resigned and a matron was engaged to take their place. This fresh arrangement was marked by the resignation of several of the older nurses, but at the annual general meeting the other day the hope was expressed that there would be no difficulty in the future in obtaining the requisite number. A system has now been established by which nurses can visit patients of moderate means or cases where the constant presence of a nurse is unnecessary. The great trouble in connection with the Home is scarcity of money, and it was suggested that some of the £4,000 which the York Charity Commissioners possess might be devoted to the benefit of the York Home for Nurses, which works entirely amongst the poor of the city. One cheering feature of the report is that the nurses, by their exertions, enabled the Council to become proprietors of the Home and to pay £1,600 towards the building fund. In these circumstances we think that the York Charity Commissioners could not do better than help those who have so admirably helped themselves.

### THE SUPERINTENDENT NURSE AND THE WORKHOUSE MATRON.

The Tamworth Guardians have appointed a superintendent nurse. In the course of the long discussion which took place before it was decided to make the appointment, the strange suggestion was made that the matron of the workhouse should be selected as "assistant nurse." The proposal provoked a rejoinder from one of the Guardians that if the matron could produce a three years' certificate that she had been trained where there had been a resident house surgeon, the arrangement might be possible. But the certificate was not forthcoming, and we are glad that the superintendent nurse, who, as the Tamworth Guardians realise, is responsible to the Local Government Board, and cannot be discharged without its sanction, becomes head of the nursing staff.

### NURSES AND TOUTS.

At a suburban police court last week a person wearing the uniform of a nurse was charged with acting as a pedlar without a certificate. Her reply to the charge was that she saw an advertisement for nurses to introduce a new kind of soap, but when she was engaged with others she never agreed to become "a licensed hawker," nor was it explained to her that she was required to carry out such duties. The bench discharged her with a caution. It is a pity that she was not asked to produce her credentials as a nurse; but in any case, it may be worth while to point out that nurses who undertake to introduce a new kind of soap, or in any other way to act as touts for tradesmen, merit severe condemnation.

### A DEFICIENCY AT COVENTRY.

It is satisfactory to observe that the first engagement of Mr. A. E. W. Mason after his election to the House of Commons as member for Coventry, was to preside at the annual meeting of the Coventry District Nursing Association. Mr. Mason, who in spite of political engagements and his work as a novelist, finds time to give close attention to the work of the district nurses in Coventry, made an excellent speech. He dwelt specially upon the fact that the increasing prosperity of the city he represents in Parliament means an increasing chance of accidents in all the industries that are carried on, and urged that, if only for that reason, it was better that the Nursing Association should keep pace with the extension. The report, which was submitted to the meeting, showed that a donation had been received from the committee of the Cycle Parade, but we scarcely think that the subscriptions, which amounted to £371, the net result of the financial operations of the year being that there is a balance due to the bank of £38, indicate adequate support of the movement on the part of those engaged in the principal industry in Coventry, whether masters or men.

### THE IMPERIAL MILITARY NURSING SERVICE.

We are officially informed of the following changes in Queen Alexandra's Imperial Military Nursing Service. On their return from South Africa, Miss M. E. Harding, sister, has been posted to the Military Hospital, Curragh; Miss E. J. Martin, sister, to the Military Hospital, Hounslow; and Miss A. Nixon, sister, to the Charing Cross Hospital for a course of two months' postgraduate study. Miss E. M. Todd, sister, has been transferred from Connaught Hospital, Aldershot, to the London Hospital, for a course of two months' postgraduate study. Miss A. A. Murphy, sister, has been transferred from the Military Hospital, Hounslow, to the Royal Victoria Hospital, Netley. Miss M. Antrobus, staff nurse, has been transferred from Queen Alexandra Military Hospital, Millbank, to the Royal Victoria Hospital, Netley. Miss M. E. Brewer, staff nurse, has been appointed to Cambridge Hospital, Aldershot. Miss M. Darvill and Miss E. A. Harvey, staff nurses, have been appointed to the Queen Alexandra Military Hospital, Millbank.

### A NEW NURSING HOME AT BLACKHEATH.

We understand that Kidbrook House, 78 Shooters Hill Road, Blackheath, is shortly to be opened as a nursing home for medical, surgical, and maternity cases, under the auspices of Miss Maud Marks and Miss E. L. Underhill, for many years respectively assistant secretary and matron of the Miller Hospital and Royal Kent Dispensary. The house, by its construction and situation, is particularly well suited for the purpose for which it is in future to be used. With an up-to-date operating-room and all modern accessories, it will, no doubt, be greatly appreciated in the locality. The list of references permitted would suffice to justify confidence in the ladies who are responsible for the undertaking, but their own long connection with the Miller Hospital and their proved capacity for the work are an even better guarantee that the Kidbrook House Nursing Home will be conducted on sound principles and in the right manner.

### QUEEN'S NURSES AND THEIR PATIENTS.

At the annual meeting of the Northampton Victoria Nursing Institution last week, the Chairman, Mr. F. Thornton, mentioned two instances of the gratitude of the patients of the Queen's nurses. One was that of a girl working as a shoe operative who sent half-a-crown—which had been given to her—as a token of her thankfulness for the attention which her brother had received during a serious illness. The other was the case of a daughter's gratitude. In December she sent £2 to the Institution, and the Chairman stated that more than ten years previously her mother had been nursed in her last illness by a Queen's nurse. Since her death, the daughter, who was also at one time a shoe operative, has forwarded several contributions, expressing the wish that other sufferers may have similar care and attention. Such incidents as these speak volumes for the admirable manner in which the Queen's nurses at Northampton fulfil their duties.

### NURSES' MISSIONARY UNION.

The first open meeting of the Nurses' Missionary Union was held last week in the class-room of the Nurses' Home at St. Mary's Hospital, Paddington. The President—who has just been elected unanimously by the nurses themselves—Miss Medhill, matron of the hospital, took the chair and introduced the speaker, Mrs. Rowland, of the Church Missionary Society, who has worked in Japan for many years. Mrs. Rowland gave an interesting account of the Japanese women, and having been a nurse herself, quite understood how nurses feel after a day's work. Accordingly, she just stated a few facts, told a few stories and concluded by appealing to the Missionary Union to remember her and Japan in their prayers. A vote of thanks was proposed by Nurse Beale, and seconded by Nurse Green. There were forty-six nurses present, and all thought that the meeting was a great success.

### A SATISFACTORY POSITION AT KINGSTON.

It was in allusion to the Home in connection with the Kingston-on-Thames Nursing Association that a patient in writing lately described his stay in the institution as "the happiest illness ever experienced." At the annual meeting the other day it was stated that although the institution is still slightly in debt to the treasurer, the report is otherwise exceedingly satisfactory. As many as 551 cases were nursed in the twelve months, necessitating 16,303 visits. In addition to these, the district nurses found time to collect donations for the Association, and by their energy substantially increased the funds. The Committee acknowledged their indebtedness to other helpers besides the nurses. A doctor gave his services free to the staff; a solicitor did the same; a lady patient nursed in the Home had caused to be built at her expense a covered way between two of the houses in Birkenhead Avenue; another lady had started a Guild of Workers to assist the nurses in their districts; and some of the local medical men had given amongst them a steriliser for use at the surgical home. Evidently the inhabitants of Kingston-on-Thames look upon their Nursing Association as worthy of all the assistance they can render it, and but a little further monetary help will enable it in 1907 to start the year with a clean balance-sheet, or even with some pounds to the good.

### THE EXAMINATION OF THE CENTRAL MIDWIVES BOARD.

The list of successful candidates who passed the examination of the Central Midwives Board on February 6, shows that 367 out of 486 passed, the percentage of failures being 24.5. Among the successful candidates were several nurses from Guy's Institution, Bristol General Hospital, St. Mary's Hospital (Manchester), West Derby Union Workhouse Hospital, the Eastern District Hospital, Edinburgh, Kensington Union Infirmary, as well as many trained at the General Lying-in Hospital, Queen Charlotte's Hospital, the Maternity Charity (Plaistow), Clapham Maternity Hospital, City of London Lying-in Hospital, the East End Mothers' Home, Liverpool Lying-in Hospital, the Royal Maternity Hospital, Edinburgh, and the Salvation Army Maternity Hospital.

### VILLAGE NURSING IN NORTHUMBERLAND.

The Duchess of Northumberland recently entertained a large number of members of the Northumberland County Nursing Association at Alnwick Castle, referring in her speech at the annual conference to the interest taken by the late Lady Grey in the organisation. It is now nine years since the Association was formed, and the last year's report shows that the work is carried on in 49 districts, the number of nurses employed being 67. Of these 51 are certified midwives. The county superintendent paid 238 visits to nurses, attended four public meetings, and 17 committee meetings; held 138 interviews, and wrote 3,310 letters—an excellent record of energy. The receipts exceeded the expenses by £132, but as the former include a balance of £343 brought forward, the financial result of the operations cannot be described as satisfactory. The need of maternity nursing in the county was strongly insisted upon at the conference.

### THE NURSE AND THE TRAMWAY COMPANY.

According to the eighth annual report of the Northfleet Diamond Jubilee Samaritan District Fund, the visits paid by the nurse—Miss Webb, who was the recipient of the Gravesend Hospital Gold Medal last year—numbered no fewer than 3,843. She works entirely under the directions of the medical men and the Committee and is supplied with all surgical and nursing requisites, as well as a bicycle. Her labours are greatly lessened owing to the generosity of the Electric Tramway Company who grant her a free pass over their system.

### SHORT ITEMS.

There were a hundred applications for the post of matron at Rosehill Children's Hospital, to which Miss Du Cane has just been appointed, and eighty-eight for the post of matron of the Victoria Cottage Hospital, Maryport, Miss Dudley being selected.—Miss E. Andrews and Miss M. O. Lindsey have been appointed nursing sisters in Queen Alexandra's Military Nursing Service for India.—At the annual meeting of the Torquay District Nursing Association it was reported that 385 cases were attended, and that 15,772 visits were paid by the nurses, and that an anonymous donor had wiped out the debt of £22 2s. 7d. on the year's working.

### The Nursing Outlook.

"From magnanimity, all fears above;
From nobler recompense, above applause,
Which owes to man's short outlook all its charm."

### THE RECREATIONS OF NURSES.

Few nurses fail to realise that contention to the uttermost as set forth in Browning's lines, "Let a man contend to the uttermost for his life's set prize, be it what it will," is an absolute necessity in their profession. They have started upon no easy task, and they find it needful to bring all their powers of mind and body to bear upon their work so that they may produce their best.

The sentiment of doing good and making others happy is a right and proper one for a nurse, but if it stays in its sentimental element and refuses to develop practically, her "life's prize" will never be won, and she will have fallen lamentably short of the mark. To produce and maintain her best she must be at her best, both mentally and physically. It is therefore our object to say something about the recreations which so largely assist in the maintenance of fitness.

Human nature abhors monotony, but it has been accused, on the other hand, of becoming enamoured of that which is ever productive of change and excitement; be that as it may, we cannot ever hope to move far beyond the ordinary routine which no doubt contains—could we but be induced to perceive it—a wholesome prescription of both sedatives and tonics.

The ranks of nurses are so numerous that these must perforce include many classes and types of people; but their work is one, and their requirements similar with regard to recreation, change, and rest. Nursing has a habit of becoming monotonous, especially in its earlier stages, because regular routine must of necessity be rigidly adhered to.

Then comes the need for the all-important tonic, and, as a rule, the dose produces the desired effect, and the recreated nurse comes back fresh and cheery to her beloved patients. As there may be many medicines good for the same disease, so there may be many diversions good for the weary brain and body; yet the best should be chosen, so that the surest and most lasting effect may result.

What are the best recreations for the nurse? Those which take her away from even thoughts of sickness must rank highest. There is the popular wheel for the cyclist. What can be more invigorating than a run along a good road between hedges?—not houses—and these roads are not hard to find, for trains are frequent and easy of access to the enthusiast who will take her bicycle a few miles by train, till she gets beyond suburban territory into the open country. The difficulties of space and position often debar the chance of tennis, but many of our hospitals are well provided in this respect, and keep courts in good condition for the most energetic to display their zeal and maintain their proficiency in this excellent sport. Those who do not enter into any of these recreations because they are "too tired" would really often feel less tired if they could shake off the feeling and enter heartily into the fun which, though perhaps using some of their physical energy, yet rests the mind, and so reacts on the body.

One most excellent exercise, suitable for summer or winter, is swimming. The water has a fascination for most nurses who realise the necessity and comfort of frequent ablutions; but, apart from this, the needful movements are splendid for expansion of the chest and the exercise of the muscles of the whole body. Where possible, this should be a recreation, and we have no doubt that the swimmer benefits immensely in many ways.

Photography, too, can now play an important part in nurses' recreations. At Guy's Hospital the nurses have a society, the members of which produce some excellent work. Slides from plates taken by nurses have been prepared, and exhibited to an appreciative audience of fellow-workers. This science—for so it may now be called—is worth cultivating, because it does not entail too much energy, and is of absorbing interest.

Literature, of course, always holds its own, and has unsolicited devotees in every profession and employment. Nurses are not as a rule at all below the mark in this respect. If they can be arranged amongst the nurses, debates are good, because they take the thoughts and the conversation from the one groove in which it is dangerous to remain if the mind is to be kept fresh and broad. Outside and current topics should be chosen, and the discussion necessary will give the impetus required to keep up interest then and after.

Gymnastics in these days are more than ever to the front, and certainly from their remedial point of view they are excellent. Why should not our nurses have their gymnastic classes purely from an exercise point of view? Acrobatic feats—which the uninitiated often suppose gymnastics to consist of—need play no part, and the regularity and rhythm of the movements are splendid training for the muscles, they also tend to develop a methodical and concentrative mind.

Any interest which really provides the nurse with suitable relaxation when off duty, and prepares her for fresher and more vigorous attention on her return, ought to be considered a recreation. Therefore we commend all those which not only give genuine pleasure while they last, but prepare for better performance of duty, and prevent the narrowing of the mind often occasioned by a too close application to one sphere.

### Abdominal Surgery.

By Harold Burrows, M.B., F.R.C.S., Assistant Surgeon to the Seamen's Hospital, Greenwich, and to the Bolingbroke Hospital, Wandsworth Common.

(*Continued from page* 303.)

### COMPLICATIONS OF HERNIA.

The chief complications of hernia are:—(1) Irreducibility, (2) obstruction or incarceration, (3) strangulation.

### Irreducible Hernia.

An irreducible hernia is one whose contents cannot be returned into the abdomen. The irreducibility may be due to the narrowness of the neck of the hernia, and in this case may soon pass on to strangulation; more often it is attributable to the contents of the hernia being adherent to each other or to the wall of the sac. Such adhesions are not likely to form unless the hernia has been neglected; and the complication is most frequently seen in long-standing hernias, in which pressure from an ill-fitting truss or other cause has resulted in the hernia becoming inflamed. The irreducible portion is omentum more often than intestine.

Once a hernia has become irreducible it is sure to give rise to trouble. It is liable to become repeatedly inflamed and painful, and is no longer amenable to the application of an ordinary truss. In addition, owing to the fact that an efficient truss cannot be worn, an irreducible hernia will almost certainly follow the usual course of untreated hernias and grow larger and larger, and thus cause ever-increasing annoyance to the patient. Moreover, a hernia in this condition is especially apt to become obstructed or strangulated.

The happiest treatment is operative, although a complete cure is not to be expected, owing to the impossibility of remedying the large defect in the abdominal wall which these old standing hernias occasion. The benefit derived from operation in these cases is that it enables the patient to regain control of the hernia by means of a truss, and so relieves him from the dangers and distresses which are inseparable from an unreduced hernia. If, on account of the patient's age or for other reasons, an operation cannot be done, treatment must be directed towards protecting the hernia from injury and preventing it from getting larger. These objects can be secured in part by means of an instrument known as a cup truss. This truss has, in place of a pad, a hollow cup, which fits accurately over the hernia. Such a truss requires very accurate adjustment and clever workmanship, and it is difficult to obtain one that is quite satisfactory in any particular case. At the same time, whether a good cup truss can be obtained or not, a constant watch must be kept for the earliest symptoms of obstruction or strangulation.

### Obstructed Hernia.

A hernia is said to be obstructed or incarcerated when the intestine which it contains has become blocked by a collection of undigested food, or fæcal material. The condition is most commonly seen in old irreducible hernias, and especially those which contain large intestine, for the contents of the bowel are less fluid in this part of the intestinal tract. Umbilical hernias in old people are especially liable to become obstructed, both because they are very apt to become irreducible and because they commonly contain large intestine. The chief cause of obstruction of a hernia is constipation, and this is not difficult to understand. For intestine that is protruded into a hernial sac is not in a favourable position to perform its peristaltic action with efficiency, and if through neglect of the bowels this intestine becomes occupied by a mass of hardened fæcal substance, it is quite possible it may be unable to expel such a mass. Another less common cause is the devouring of large quantities of indigestible material which presently forms a hard mass within the loop of protruded bowel, and so leads to obstruction in the same way as that just described.

It follows that patients who have irreducible hernias ought to be particularly careful to eat moderately, avoiding foods that leave a large indigestible residue in the intestine, and to avoid constipation. If in such a patient constipation comes about, and cannot be relieved readily by means of an injection, obstruction of the hernia should be suspected. And the suspicion will be greatly strengthened if the patient begins to vomit, and the hernia is found to be hard and tender; although the absence of these last-mentioned symptoms does not necessarily mean that the hernia is not obstructed. As soon as a suspicion of hernial obstruction arises the patient's medical attendant should be called in, for the condition is one which is full of danger unless it is treated in time.

The treatment is to give rectal injections, usually of soap and water. If these do not bring relief, attempts may be made to reduce the hernia. These will probably fail. In any case, "taxis," as the manipulation of a hernia to reduce its contents into the abdomen is called, should not be applied by anyone but a qualified medical practitioner, because unskilful attempts may do a great deal of harm, and may kill the patient. If rectal injections fail, and the hernia cannot be reduced, an operation will be necessary. The objects of an operation in this case will be to open the sac of the hernia, reduce the bowel into the abdomen, where its peristaltic movements are no longer hindered, to remove the hernial sac, and to close the opening in the abdominal wall through which it has escaped.

### Strangulated Hernia.

Unlike the complications of irreducibility and obstruction, both of which are most commonly seen in hernias of long standing, strangulation more frequently occurs in hernias which have seldom been

"down." What happens in strangulation is this: a piece of intestine slips into a hernial sac whose neck is so narrow that not only is it impossible to return the protruded bowel into the abdomen, but the circulation in the imprisoned portion of bowel is hindered and ultimately arrested.

It has been pointed out already that repeated descent of a hernia leads to stretching of the neck of the sac, and bearing this in mind it is easy to understand that a neck which has not been stretched is more likely to cause strangulation than one which has been dilated by frequent descent of the bowel.

The onset of strangulation is usually accompanied by pain in the hernia and in the region of the umbilicus, and perhaps there is a feeling of nausea. The hernia itself becomes tender and hard, and cannot be reduced, and an expansile impulse is no longer transmitted to the hernia when the patient coughs. If the strangulation is allowed to continue two grave results will follow—assuming that a portion of bowel is in the sac. In the first place, intestinal obstruction is caused; this soon leads to vomiting, and if the case is neglected the vomited matter becomes fæcal in character. Secondly, the circulation of blood in the imprisoned portion of bowel being arrested, gangrene of the bowel will ensue, the walls of the intestine will give way, and the contents of the bowel will escape into the hernial sac and into the general peritoneal cavity, setting up acute septic peritonitis, which under these circumstances is almost always fatal. From this it will be seen that strangulation is a fatal complication of hernia unless the portion of intestine concerned is speedily released. As in so many other abdominal complaints, the patient probably will recover if correct treatment is available at once, whereas he is nearly sure to die if treatment is too long delayed.

The treatment consists in releasing the imprisoned bowel at the earliest possible time. This may occasionally be achieved by taxis, which must be of the gentlest, and must only be applied by a medical man for similar, but even more cogent, reasons than those mentioned already in connection with obstructed hernia. If taxis fails, the only reasonable hope lies in operation, and preparations must be made at once for carrying this out. In the meantime the patient should lie on his back in bed with a couple of pillows under his knees, and his head and shoulders well supported with pillows so as to flex the body and relax the abdominal walls. An ice-bag, or, failing this, pieces of linen wrung out of cold water, should be kept in contact with the hernia. In other cases, and especially in young and robust patients, a hot bath is serviceable. The water must be as hot as the patient can bear, and the taxis is performed while he lies in the bath. By these measures it sometimes happens that a second attempt at reduction by taxis is successful. If not, operative treatment, which is similar to that already described as the radical cure of hernia, must be proceeded with. The after-treatment has no especial features, and is similar to that of any simple laparotomy. It often happens that a truss has to be worn after the operation wound has healed, and this is usually the case with femoral hernias. While waiting for the truss the patient should not on any account be allowed to get about without some support to the wound, or the hernia may speedily recur. In the case of a femoral or inguinal hernia, a pad of wool firmly applied to the region of the operation wound by means of a spica bandage may be used as a temporary substitute for a truss.

### Other Complications.

These are reduction *en masse*, interstitial hernia, rupture of bowel in a hernia, rupture of a hernial sac with protrusion of the bowel. Reduction *en masse* and interstitial hernia are both caused by forcible and usually misguided efforts to reduce the hernia either by the patient himself or his friends. In reduction *en masse* the sac is torn away from its attachment and pushed together with its contents into the abdominal cavity; and in interstitial hernia the sac with its contents is merely displaced underneath the abdominal muscles. It is clear that in neither case is the hernia reduced, correctly speaking. If, as is often the case, the forcible efforts at reduction have been made on account of obstruction or strangulation of the hernia, the symptoms of these complications will continue in spite of the disappearance of the tumour. If, therefore, the symptoms of obstruction or strangulation persist after reduction has apparently been performed, suspicions of reduction *en masse* or interstitial hernia ought to arise, and preparations should be made forthwith for an emergency operation.

There is not much to say about the remaining two complications. Rupture of the bowel in a hernial sac is not a very rare injury, and it is easy to imagine how a blow, which need not be very hard, upon a hernia, and especially upon an irreducible hernia, may rupture the intestine which it contains. The symptoms are those of perforation of the bowel, a matter which will receive attention in a subsequent article.

Rupture from violence of the hernial sac and its coverings, so that the bowel protrudes through the skin, is most likely to occur in old and extensive hernias where the tissues have become greatly thinned by pressure. It probably will cause septic peritonitis by allowing the direct entry of germs into the peritoneal cavity. The fact that the accident may be unattended with pain occasionally leads to fatal delay of the necessary operative treatment. I well remember an old woman whose neglected femoral hernia one day burst, so that the bowel escaped on to the surface of her thigh. As the protruded intestine was insensitive, and the old lady did not know what it could be, she was just about to snip it off with a pair of scissors when her daughter, arriving on the scene, fortunately dissuaded her from the act.

As soon as rupture of a hernia is discovered a warm antiseptic compress should be applied over the protruded bowel and a wide area around, and preparations made at once for the operation, which is the only treatment. In the meantime the patient must lie quite still, with the knees and body flexed, as in strangulated hernia; and steps must be taken to lessen the shock, which may be severe.

### The Nurses' Clinic.

### OPHTHALMIC OPERATIONS.

In eye operations the rules, with regard to antisepsis and asepsis, are the same as for general operations.

The operating room is made surgically clean, the table so placed as to get a side light, and not an overhead one, which is liable to throw shadows on the eye and confuse the surgeon. The table (if in a private house) should be first well scrubbed with soft soap and soda, and when dry covered with a couple of blankets, a rubber sheet, and then a clean cotton sheet. The pillow is covered with a mackintosh and a sterile towel, which prevents the unpleasant chill that would be caused by putting the patient's head on the cold mackintosh. A small two-story table should be placed opposite to the window, with the surgeon's instruments and dressings, solutions, etc. A second table for solutions and dressings is preferable. The anæthetist's table should be provided with the usual things—namely, anæsthetic mask, gag, tongue forceps, hypodermic, brandy, basin, swabs, vaseline, and a battery in case of need. The room or theatre should be at a temperature of 80° F.

In preparing eye instruments the greatest care must be exercised by the nurse not to blunt the edges, as the instruments are very small and delicate. Larger instruments such as speculum, tenotomy hooks, needle-holders, etc., can be boiled in soda solution, or sterilised by steam. The delicate knives, cataract knives, keratome and knife needles should only be dipped into boiling solution for a minute, not allowing the blades to touch anything. They are then dipped into alcohol, rinsed in sterile water, and placed in trays ready for use. Or they can simply be dipped in absolute alcohol and dried just before using. Small gauze sponges are used as swabs, and these, with the bandages, strips of gauze, etc., should be sterilised, preferably by steam. The ligatures, generally of silk, are in bottles sterilised ready for use. Dressing-trays, bowls, etc., should be boiled for ten minutes before being used, and dried with sterilised towels. The solutions should be sterilised by boiling before use. Hands are sterilised as for general operations, great care being given to the nails.

For major operations the patient is prepared as for a general operation, but many eye operations are performed under a local anæsthetic as cocaine, eucaine, or holocaine, in which cases no general preparation, such as cathartic and enema, would be resorted to; though in all cases it is most necessary to keep the bowels well open.

In major eye operations the greatest care must be taken to prevent the patient taking any food before the operation, as vomiting is specially harmful in eye cases, as the violence of the vomiting may sometimes even extrude the contents of the eye. At the first sign of vomiting the patient should be kept lying perfectly flat, all pillows being removed, and a mustard-leaf applied to the stomach. Small drinks of warm or hot water are generally effectual in arresting the sickness, or sucking in; though the former remedy is most satisfactory.

If the operation is near the eyebrow, this must be shaved off before the patient comes to the theatre. A rubber cap is put on the patient to prevent the instruments or surgeon's hands from coming in contact with the patient's hair. Then the outer surface of the skin is washed with soap and water, the edge of the lids and eyelashes getting special attention. The skin should then be washed with solution of bichloride of mercury 1 in 5,000. The lids are next turned and wash with warm sterile water or boric solution.

Some surgeons prefer having the eye well washed the day before and left with an aseptic compress till the operation.

Local anæsthesia is begun ten minutes before operation, when the nurse drops the solution into the eye. In dropping solution it is to be remembered that it should be dropped into the outer end of the eye and the tear-duct closed with the finger, or the solution may be absorbed through the tear-duct and alarming symptoms arise. Before operation, a drop of cocaine should be instilled in the sound eye, which must be covered with a pad held on by adhesive plaster.

After most eye operations the patient is kept in a darkened room, and lying nearly flat in bed. Light nourishing diet, and no worry or excitement allowed. For the first twenty-four hours, at least, a nurse should always be at hand to attend to every want, so that the patient has no reason to sit up or get out of bed, and with children special caution must be used to prevent them pulling off their bandages. If after operation a wound is persistently painful and the eyes running, it may be caused by infection, in which case special attention must be exercised. The eye should be looked at three or four times a day, well bathed with hot boracic solution, and atropine instilled. Sometimes the surgeon may cauterise the eye, so the nurse should always have the cautery ready if required. Deep infection of the eye is dangerous, cases of death from meningitis have been known through the infection extending to the brain. In infected wounds the nurse will have plenty of work in making the hot application, cleaning the eyes, keeping stock dressings, etc. always ready. Should hæmorrhage into the eyeball occur it must be reported immediately; little can be done as a rule, and generally the sight is lost.

After operations, especially senile cataract and iridectomy, occasionally patients suffer from shock or delirium, leading to acute mania; this occurs sometimes when both eyes are bandaged and the patient is in utter darkness. It does not always set in directly after operation, perhaps not for four or five days. Freeing one eye will sometimes give entire relief, though it may be necessary to remove all bandages. Of course this would only be done under doctor's orders; when very violent, opiates will be given, and, of course, the patient must not be left for one minute alone.

The various shields, bandages, etc., used for eyes cannot be described in a short article like this, and indeed can be thoroughly learnt only in the wards of an eye hospital.

Before concluding I must add a few words as to what steps to take in cases of emergency such as burns, injuries, or poisoning, till medical assistance can be procured.

*Burns*, caused by lime.—Do not let water touch the eye, but take a clean handkerchief, gauze, or linen—I mean with oil, castor oil, butter, or any simple oily substance—and clear all the lime out of the eye, turning the lids to see that no particle remains. Sugar water or vinegar are useful, as they neutralise the lime. Cold compresses should then be applied till medical assistance can be called.

*Acids.*—In cases of burning by acid, wash the eye directly with an alkaline solution. Mix either borax, soda, or bicarbonate of potash with water, milk, or plain water, to dilute the acid as much as possible. The lids should then be greased and cotton applied till aid arives.

*Steam, boiling fat, hot cinders, etc.*—First remove any foreign body, then smear the lids with vaseline or simple oil or ointment, and a compress of weak carbolic lotion (1 per cent.). Where there is shock, administer stimulant.

*Injuries by Violence.*—Cleanse the eye and apply compress of weak boracic lotion. If the eyeball is injured put one drop of atropine sulphate (1 per cent.) and cover up with clean linen bandage till surgeon arrives.

### THE NURSES' CLINIC—*Continued.*

*Infection from Pus.*—If, while dressing and cleansing a patient's eyes who is suffering from gonorrhœal ophthalmia, some of the pus should get into the surgeon's or nurse's eye, wash immediately with solution of bichloride of mercury (1 in 5,000). Then drop nitrate of silver (2 per cent.) two or three drops, into the eye. Iced cloths may be applied to allay the pain and irritation.

*Poisoning from Atropine.*—The throat is dry, giddiness and difficulty in swallowing, pupils dilated and fixed, eyelids red and swollen, the face flushed, and the pulse rapid, convulsions, delirium ending in coma and death. Administer stimulants, as coffee, whisky, and strychnine, and drinks to moisten the throat and mouth.

Hypodermic injections of morphia as an antidote, and caffeine and camphor to strengthen the heart. Hot bottles to extremities, and artificial respiration when stupor sets in.

*Cocaine.*—Symptoms: Weakness, dizziness, rapid irregular pulse. Lay the patient down flat, undoing neckbands, belts, etc. Give brandy and coffee by mouth and camphor or strychnine by hypodermic.

### Incidents in a Nurse's Life.

### IN A UNION FEVER HOSPITAL.

It was a bitterly cold night in December when I found myself standing on a little platform in the North of Ireland, bound for a union fever hospital some twelve miles away, as a temporary night nurse. Having alighted from the railway carriage—the midnight mail—I looked about to see if there was anyone sent to meet me, and discovered a car in waiting. A drive of twelve long miles in the small hours of a bleak winter's morning—it was 1.30 A.M.—on an Irish jaunting-car is not very tempting, as any nurse who has had the experience will admit. Still it had to be, so I determined to make the best of things. The driver made numerous apologies for his horse—a badly fed, ill-kept animal who could accomplish no more than four miles an hour—and parts of the road were so bad that at times I fully expected to be thrown off the car altogether. After somewhat more than three hours of rail, and another three hours of driving, I arrived at the great black gates which characterises almost every Union in Ireland. Here, after much jingling of keys, I was admitted by that important individual the porter, the carman was ordered to deposit my traps and begone, whereupon, having securely locked the great iron gates, the worthy official went off to inform the master of my arrival. Then I was escorted through several passages, doors were unlocked and locked again the moment we had passed through, then we traversed a great dark yard, yet another door to be unlocked and locked as quickly, when we found ourselves in a trim little garden in the centre of which stood a neat brick building which I rightly concluded was my destination. The door stood open and a ray of light and warmth issued forth, seeming to suggest a sense of comfort and homeliness which, from the appearance of the other sections of the building I hardly expected to find.

Just as we crossed the threshold, the matron—or nurse in charge as she is sometimes called—came forth to welcome me. Such a cheery homely woman! and so kind and sympathetic over my long cold journey, as she ushered me into her cosy little sitting-room where a bright fire was burning, a comfortable chair was placed temptingly in front of it, into which she placed me "to thaw," as she expressed it, while she went off to finish something she was doing for a patient. Later I found a charming meal awaiting me, and nurse had joined us. The table, which was laid when I arrived, was pulled up close to the fire, and nurse having made tea, we were both soon enjoying the warming beverage, I also despatching a chop with the greatest relish, together with quite an amount of hot buttered toast, which seemed to have been made specially for me. Often during our meal I found myself thinking what a kind thoughtful soul nurse was. There was every proof of her goodness in the wards, where she took me to show me the patients and give me directions, while she went off duty, having been on for more than forty-eight hours without a break. There were two wards on the ground floor, each containing six beds; these wards were reserved for the female patients, while upstairs were two wards with ten beds for male patients. The patients at the time were five girls and a tiny boy of five years, all suffering from diphtheria of the worst type. They were all in one of the wards where there were neither shutters nor blinds on the windows, but hanging curtains of green calico saved the poor sufferers' eyes from the bright wintry glare; little screens, fashioned from small folding clothes-horses covered with Turkey red, were placed round the beds near the door to guard against draughts; a fire burned quietly in the grate, and everything looked spotlessly neat and clean; moreover, there was a satisfied look about the little patients which it was good to see. I discovered afterwards that the window-curtains and the screens and the other little comforts for the patients, were all the result of nurse's thought and skill. Having given me directions as to the treatment of the little people, nurse went to take her well-earned rest, while I made poultices which were just due, and gave some warm milk to the patients; I was taking the drinking vessels to the kitchen to wash them when I was arrested by a noise like a continuous peal of thunder which seemed, as it were, quite beside me. It was now a quarter past six A.M. and this noise had been going on for fully five minutes, when I remembered that it was time to call the wardmaid, so I repaired to her room in the hope that she would be able to enlighten me as to the cause of the unearthly noise. I was highly amused when she told me that it was only the tramps pumping up the water for us, I discovered afterwards that they came every morning at 6 A.M. and pumped until 7 A.M., this much work being exacted in return for their night's lodging and breakfast, which they got on the completion of their task. The doctor came at 9 A.M., greeted me very kindly, said he was glad I had come as his nurse was half dead and he could not get a second nurse although he had wired for one two days ago to three different hospitals. He seemed quite proud of nurse, and spoke of her as the best nurse he had ever met, in which opinion later I also agreed. Having seen the patients and given some fresh instructions regarding their treatment, the doctor, as soon as he had scrubbed and disinfected his hands, departed. He had not been gone more than an hour when the master came to announce that he had instructions from the doctor in a neighbouring district to send the fever ambulance for two patients who were also suffering from diphtheria. I procured the things necessary for the ambulance, saw that the wardmaid who had to accompany it to take charge of the patients was ready, and then set about preparing for their arrival. The doctor had said nothing about the sex of the patients, so there was nothing to do but prepare beds in both male and female wards, and, as the fires were already set, I had only to put a light to them; I had just finished and was going to the kitchen to fill some hot bottles when I encountered nurse. As it was only twelve o'clock and she had not gone to bed till after six, I began to reproach her for stirring so early, but she said she was scarcely in bed before she was sound asleep, and she had not awakened till now, thoroughly refreshed, although the maid had been in and left a cup of tea beside her. Personally, I should have thought that the rumble of the ambulance as it left the yard would have roused the soundest sleeper; but she had never heard it, and was quite surprised when I told her that we were getting in two more patients. I had fully intended that after her long vigil she should have had her breakfast in bed, but my good intentions were not destined to be fulfilled, so all I could do was to have the meal prepared ready by the time she was dressed.

When the ambulance came back we found it to contain a boy of fifteen years and a girl of nine, brother and sister. Both seemed as if a hot bath and plenty of soap would have been a great improvement, but, as they were very bad, we had to defer it until they were in bed, when they were treated to a bed bath, to which they did not take at all kindly at first, the little girl telling me that her mother would not wash them since they got sick as she said that it would give them cold. After that first wash both brother and sister began to have less dread of soap and water until finally they grew to like it.

Almost every day now the ambulance was being requisitioned to convey fresh cases for us, until instead of six patients we could count sixteen, all suffering from diphtheria. Like the little brother and sister, all the other patients who were admitted had the same horror of soap and water. One patient, a woman, went so far as to tell me that I should be the death of her, because I washed her—and she very badly needed it, too. She did not die, however, but lived to laugh at her folly and appreciate the value of soap and water. It was strange the way the people shunned us whenever we went out for a walk. Women standing in their doorways chatting, would, the moment they saw one of us approaching, scamper off to their own houses, and by the time we reached that particular place not a soul was to be seen, every door was shut and probably barred also, and perchance if one could only see it, the inmates of those little homes were at that moment on their knees praying for preservation from the "faver." Once I met two old women who saluted each other as I passed them with: "God protect us, 'tis the nurse from the Faver House," both at the same time crossing themselves and keeping to the farthest corner of the road lest I might infect them. No person ever came our way who could avoid doing so.

The doctor, who was most attentive and kind to his patients, was very proud, too, that out of his sixteen cases there was not a single death, although some were very bad and neglected when admitted. The gratitude of the patients on their recovery was good to behold, the doctor's praises were sung many times a day, and no end of blessings invoked on him and his family. The nurses also were not forgotten.

When after thirteen weeks the doctor one morning told me that I should soon be going, I heard the decision with genuine regret, for the doctor, the nurse, and the patients, one and all, seemed to have grown as dear to me as if I had laboured amongst them all my life. And as I started two days later on my long drive to the station, though sad, I felt the richer for that sojourn in the little fever hospital away in the wilds, for I carried away with me many happy memories and the friendship of a woman whom I had learnt to admire more, both as a nurse and as a woman, every day I spent with her.

### Central Midwives Board.

A meeting of the Central Midwives Board took place on Thursday last, at which there were present Dr. Champneys (Chairman), Mr. Ward Cousins, Dr. Dakin, Mr. Fordham, Mrs. Latter, Miss R. Paget, Sir William Sinclair, Miss Wilson, and Mr. Parker Young.

### Proposed Official Handbook for Midwives.

Several letters were received from the examiners of the different centres pointing out the great want of uniformity in the teaching of candidates for the examination and the defective training of many. Dr. Fothergill, of Manchester, wrote suggesting that an official handbook for midwives be issued by the Board for the guidance of teachers and candidates. The Board felt that any question of laying down lines for teaching must come up at the meeting for the revision of the rules, and in the meantime they decided to call a meeting of examiners, with Dr. Champneys in the chair, to discuss the question of an official handbook and any other business which it might be fit to include in the agenda.

### Consideration of Letters.

A letter from Mrs. Heywood Johnstone as to the difficulties experienced by women of the working class when appearing before the examiners was also read, and all the members of the Board who had been present at the examinations unanimously testified to the care and patience exercised by the examiners in such cases. Regarding a letter from the Clerk of the County Council asking whether the midwives in a certain London private maternity hospital, visited almost daily by a paid medical attendant, should be considered exempt from inspection in accordance with Rule E 21, the Board instructed the Secretary to reply that it was not intended that the rule should refer to private institutions, and to urge the County Council to use their influence with the Local Government Board for the removal of this rule, at whose instance it had been framed. The Secretary reported that at the February examination there had been 490 candidates (486 of whom sat for the examination), as against 471 in October; 341 of these had attended at the London centre, the rest being distributed between Bristol, Manchester, and Newcastle. The percentage of failures was about the same.

### Report of the Penal Cases Committee.

The report of the Penal Cases Committee was then received. The recommendations of the committee, which were carried, were as follows: (1) That a solicitor be employed to prepare the evidence to be brought before the Board in penal cases, and to secure the attendance of the witnesses, where possible; (2) that statutory declarations be received as evidence of the facts declared, where otherwise admissible by law; (3) that a small committee be formed for the preliminary consideration of penal cases, and to recommend the Board as to whether proceedings should be initiated. The committee further suggested that the remuneration of the solicitor should be arranged on the following basis: A retaining fee (say, twenty guineas) per annum to be paid, together with ordinary costs and out-of-pocket expenses, subject to taxation by the Board; the retaining fee to be payable so far only as the total amount of costs payable by the Board during the year falls short of the amount of the retaining fee. The committee were of opinion that it was not desirable to advertise for the services of a solicitor.

### Appointment of New Officials.

Accordingly the Board decided that Mr. Julius Bertram, who had been approached on the subject, should act as their solicitor. Dr. Champneys, Mr. Fordham, and Mr. Parker Young were appointed the committee for the preliminary consideration of penal cases. The report of the standing committee was then taken. Miss Jennie Hambling was appointed head clerk in the place of Miss Menzies (resigned). The Board passed a vote of thanks to Miss Menzies for her extremely efficient services to the Board. The following additional examiners were appointed for the London Centre: Victor Bonney, M.D.; H. T. Hicks, F.R.C.S.; Mary E. Rocke, M.D. For the Bristol Centre: W. C. Rayner, F.R.C.S.; Smallwood Savage, M.B., F.R.C.S. For the Manchester Centre: W. Kay Walls, M.B. Motlibai Hospital, Bombay, was approved as a training school, also Sheffield Union Hospital, subject to the perfecting of the syllabus. The application of Edmonton Union Infirmary was refused, and of Whitechapel Infirmary, on account of structural unfitness.

THE CERTIFICATES OF ATTENDANCE.

There was some discussion on the applications for approval to sign Forms III. and IV. (certificates of attendance on cases), and Sir William Sinclair urged that the Board should be most careful not to approve for such purposes illiterate women who would be quite unable to give the proper instruction required. On his recommendation the application of Margaret Evans, of Nantyglo, Mon, who was working without any supervision and who was only a *bona fide*, was refused. In view of the fact that the period of approval by the Board of the present list of midwives authorised to sign Forms III. and IV. expires on March 31 next, it was decided that a circular letter be sent to all the midwives on the approved list, requesting them to inform the Board whether they wish to continue to take pupils for training after March 31, on which day their present period of approval expires. Mr. Fordham moved that it is desirable that copies of all reports of committees of local supervising authorities and of county and borough medical officers and other officers of such authorities relating to midwives, or to the proceedings of such authorities in execution of their duties under the Midwives Act, 1902, be forwarded to the Board for their information; and that this resolution be communicated to the County Councils Association, the Association of County Boroughs, and all local supervising authorities. The motion was carried. Mr. Ward Cousins moved that the form of suggestions accompanying the agenda of December 14 for the guidance of the medical advisers of the local supervising authorities, respecting the duties of inspectors appointed to exercise general superintendence, be approved and issued to the local supervising authorities. It was agreed that this be preferred to the Standing Committee, as some members of the Board felt that certain of the suggestions were not suitable, especially considering that many of the most efficient local supervising authorities had already formulated sound views of their own on the whole question. A meeting of the Standing Committee for the revision of the rules was fixed for March 8, and the next meeting of the Board for March 29.

### To Nurses.

WE invite contributions from any of our readers, and shall be glad to pay for "Notes on News from the Nursing World," "Incidents in a Nurse's Life," or for articles describing nursing experiences at home or abroad dealing with any nursing question from an original point of view, according to length. The minimum payment is 5s. Contributions on topical subjects are specially welcome. Notices of appointments, letters, entertainments, presentations, and deaths are not paid for, but we are always glad to receive them. All rejected manuscripts are returned in due course, and all payments for manuscripts used are made as early as possible after the beginning of each quarter.

### Practical Hints.

**We welcome notes on practical points from nurses.**

### A DISTRICT NURSE'S PUDDING CLUB.

A GREAT problem in district nursing is the absence of suitable food for invalids and convalescent cases among the poor. I have found here in Dunstable that a "Ladies' Pudding Club" solves the difficulty. Each member makes herself answerable for a milk pudding or a pint of beef-tea weekly. Duplicate tickets are written out by the district nurse, the notice part being sent in by the patient the day before, while the ticket part is taken when the pudding or beef-tea is fetched. A rule strictly enforced is that the dish or jug must be returned by the following day. The form of ticket is as follows:—

| NOTICE. 1 | TICKET. |
|---|---|
| To Mrs. .............. | To Mrs. .............. |
| Address .................................... | Address .................................... |
| Milk Pudding or Beef Tea for | Milk Pudding or Beef Tea for |
| Name of Invalid ......................... | Name of Invalid ......................... |
| Date: For Tuesday. | Date: Tuesday. |

If milk pudding be needed, the words "beef-tea" are scratched through, and vice versa. Unless otherwise stated, the choice is left to the giver.

A tabulated list of names is kept opposite a schedule of weeks, in order to apply to the members as far as possible in turns.

FORM OF LIST. WEEKS ENDING

| | Feb. 7. | Feb. 14. | Feb. 21. | Feb. 28. |
|---|---|---|---|---|
| Mrs. Smith ... ... ... | 3 | 10 | | 24 |
| Mrs. Jones ... ... ... | 4 | 11 | | |
| Mrs. Brown ... ... ... | 5 | | 15 | |
| Mrs. Robinson ... ... | 7 | | 17 | |
| Mrs. Green ... ... ... | | 8 | | 22 |

This scheme has been worked since November 11, 1905, during which time 134 invalid nourishments have been provided by a club now consisting of 28 members.

### Presentations.

NATIONAL HOSPITAL FOR DISEASES OF THE HEART.—Miss Barber, who has resigned her appointment of matron of the National Hospital for Diseases of the Heart, London, which she has held for three years, was presented, on the occasion of her marriage to Dr. A. Tanner Cooper, with a handsome clock from the honorary physicians of the hospital, many charming gifts from the nursing staff, and a set of silver saltcellars from the maids.

WESTERN INFIRMARY, GLASGOW.—Miss Shannon, who has lately retired from the matronship of the Western Infirmary, Glasgow, was presented by the nurses with a handsome cabinet writing-desk, by the domestic staff with a case of silver fruit knives and forks and a silver gong, and by ladies representing the Nurses' Union of the Young Women's Christian Association in Glasgow with a pair of silver candlesticks.

### Appointments.

[No charge is made for announcements under this head, and we are always glad to receive and publish appointments. The information, to insure accuracy, should be sent from the nurses themselves, and we cannot undertake to correct official announcements which may happen to be inaccurate. It is essential that in all cases the school of training should be given.]

Aberdeen Dispensary, Maternity Hospital, and Lying-in Institution.—Miss Alice M. Bewlie has been appointed superintendent. She was trained at the Infirmary, Withington, Manchester, and has since been sister at Queen Charlotte's Hospital, London, and matron at the Hospital for Accidents, Somerton, Somerset.

Chadwell Isolation Hospital.—Miss B. Lewsey has been appointed sister, and Miss M. McLachlan night sister. Miss Lewsey was trained at Stepney and Poplar Sick Asylum, and has since been sister at Kensington Infirmary and sister at Stirling District Asylum, Larbert. Miss McLachlan was trained at Birkenhead Infirmary, and has since been night charge nurse at the Alexandra Hospital, Coatbridge, and sister at Woolwich Poor-law Infirmary.

Cottage Hospital, Horley.—Miss A. Horker has been appointed sister-matron. She was trained at Lincoln County Hospital, and has since, as a member of the Army Nursing Service Reserve, done work in South Africa. She has also been nurse at Monsall Fever Hospital, Manchester, and charge sister at Lavenham Infirmary. She holds the certificate of the Central Midwives Board.

General Hospital, Northampton.—Miss Mildred Tomlinson has been appointed sister. She was trained at the Royal Infirmary, Preston, and has since been sister at the Royal London Ophthalmic Hospital, Moorfields, E.C.

Infectious Diseases Hospital, Burslem.—Miss Laura Garton has been appointed sister. She was trained at Ayr County Hospital, and has since been nurse at Frazer Hospital, Hull; the Hospital for Women, Liverpool; the General Hospital, Dorking; and Haywood Hospital, Burslem.

Infectious Hospitals, Heathcote and Fosse Road, Near Leamington.—Mrs. Edith Thomas has been appointed matron. She was trained at the Royal Hants County Hospital, Winchester, and has since been nurse at Johannesburg Hospital; night superintendent in the same Institution; nurse at Victoria Nursing Home, Bournemouth; and night superintendent at West Bromwich Infirmary. She has also done private nursing.

Queen Victoria's Jubilee Institute for Nurses.—Miss Ethel Dixon has been appointed superintendent of the Hampshire Nursing Association affiliated to Queen Victoria's Jubilee Institute for Nurses. She was appointed Queen's nurse in January 1897, and has worked in that capacity in York, Darlington, Woolwich, and Swansea.

Midlothian and Peebles District Asylum, Rosslynlee, N.B.—Miss Jenny Thyne has been appointed matron. She was trained at the Royal Edinburgh Infirmary, and has been for nearly two years assistant matron at the Stirling District Asylum, Larbert.

Rosehill Children's Hospital, Torquay.—Miss Du Cane has been appointed matron. She was trained at Salisbury Infirmary and Leicester Infirmary. She has since done district nursing at Poole, been sister and then matron of the Nottingham Children's Hospital, and matron of the Cosmopolitan Hospital and Nursing Home in Venice.

Tiverton Infirmary.—Miss Violet M. Helby has been appointed matron. She was trained at the Royal Portsmouth Hospital and the Bedford County Hospital. She has since been sister at the Bedford County Hospital, and sister at Tiverton Infirmary. She has also done private nursing at home and abroad; and on three occasions she has acted as matron's *locum tenens*.

Victoria Cottage Hospital, Maryport.—Miss B. Dudley has been appointed nurse-matron. She was trained at the Royal Infirmary, Liverpool, where she has since been staff nurse and ward sister.

### Everybody's Opinion.

[Correspondence on all subjects is invited, but we cannot in any way be responsible for the opinions expressed by our correspondents. No communication can be entertained if the name and address of the correspondent are not given as a guarantee of good faith, but not necessarily for publication. All correspondents should write on one side of the paper only.]

### "OVERWORKED PROBATIONERS."

"X. Y. Z." writes : I suppose that even probationers will admit that there are two sides to every question, but in the correspondence going on just now in your columns the writers seem entirely to overlook the side of the hospital authorities and of the Matron. Hospital finances will not admit of a superfluity of nurses to take the places of those who think they ought to be taken off duty by the Matron every time they have "a cold, a headache, or a bad finger." The Matron is responsible to the Committee, the medical staff, and to the governors generally for the proper and efficient nursing of the patients. How do the nurses think this is to be done if they themselves "go sick" for every trifling ailment? "A Provincial Nurse" writes of the novelty of working a nurse who for months suffered from nervous headache and ended in committing suicide. I am sure that the Matron knew nothing of these "months" of headache, or she would have dismissed a person who was obviously unfitted for hospital work. People who suffer from "nerves" ought never to think of taking up a work which must in the nature of things cause a strain upon them. But, if she does, is it fair or just to blame the Matron? The fact is that in these days the majority of young women who go in for hospital training are such a set of fragile, neurotic, self-pitying invalids that one wonders why they go in for work which cannot be scamped even if the workers have colds and headaches. Do these nurses ever consider that matrons themselves have an immense amount of brain fag and of responsibility coming after years of hard work as nurses and sisters? yet they do not think of giving up for every ache and pain. I have also frequently known house-surgeons to be working with severe colds, "heads," and throats, but who scoff at the idea of going to bed on that account. "A Provincial Nurse" writes, "If Matrons would interest themselves in the welfare of their nurses." I maintain that there never was a time when matrons took as keen an interest in their nurses as they do now. In almost every hospital one finds she has obtained pretty sitting rooms, piano, library, tennis courts, croquet ground, and sometimes hammocks for their pleasure. But these things are all accepted nowadays as a matter of course, and without the slightest feeling of gratitude. I have at last reluctantly come to the conclusion that Matrons will "show their interest" best in these delicate self-pitying young women by dismissing them from the staff. Certainly it would be to the interest of the patients, and of those nurses who have sufficient backbone and stamina, and who have to do double duty while their companions lie in bed reading novels, and looking more or less fascinating in smart dressing jackets. Of serious illness I am not speaking. Every nurse knows that if seriously ill she will receive the most loving care and attention. I have only one thing more to say. In spite of warnings, these nurses, whom I must call "shirkers," will not take proper precautions. They will not wear warm underclothing. They will not wash out their mouths when attending foul septic cases. I am told that they are not careful about washing their hands before going to meals. In fine weather they often will not go out of doors, and in wet weather out they go, more especially if they have a tendency to cold. They will not cover any prick or scratch on their own hands till the mischief is done, and then "that tyrant the Matron" is not sympathetic, and even expects them to go on duty. Oh for the good old days of twenty years ago! Women then went into hospitals to be workers, not to be patients; they went from a genuine desire to help and benefit suffering humanity, not thinking of themselves and their aches and pains. They expected hard work, rough accommodation, and the plainest of fare, and they got what they expected. Yet one never heard a complaint nor a grumble. Can one say as much now?

### THE RESPONSIBILITIES OF A SISTER.

"M. L." writes: I should like to add my opinion to that of "H. M. E." on "Sisters' Responsibilities." I am sister of a male medical and surgical block containing 64 beds, and have three probationers, the senior nurse being in her third year and two in their first year of training. They stay with me from two to three months, and generally all are moved to other wards within one week. What a blessing a staff nurse would be at such a time, for, naturally, new nurses need more supervision and instruction the first week after their change than afterwards is necessary, and how can a sister, however energetic she may be, watch three strangers at the same time? I have held my present post about three years, and as time goes on I feel more keenly every item of responsibility in connection with my patients. I am quite sure that the strain of supervising nurses who are persistently careless and indifferent tires me more than any ward work or scrubbing of mackintoshes did in my pro.'s days. If probationers would only make up their minds to do their best always, not only when "Sister" is likely to come along to inspect, what an immense help that would be only sisters know! And also in the matter of correction; nurses should take reproval of blunders, carelessness and mistakes, as part of their training, and given for their own benefit as well as for the welfare of their present and future patients. I am afraid that quite a large proportion of nurses consider correction, which they think of as scolding, however well deserved, an unnecessary evil which must be resented, either by sulkiness, complaining at headquarters that "Sister expects too much, and is never satisfied," or, worst of all, by impertinence, and I am rather inclined to think that the last-named method is on the increase. How helpless one feels in a busy time when the nurses act so, and especially when Matron will listen to nurse's tale, and, perhaps, prevented by more pressing calls on her attention from investigating the cause of the "rub," makes peace by giving nurse a premature move on, whilst another stranger comes to be initiated. I certainly think that a sister of a large busy ward should have a staff nurse to take some share of responsibility, if only of ward work, bathing, linen stores, etc. The at present over-tired sisters then could take a more active part in the actual nursing, and carry out more directly the treatment ordered by the doctors.

### NIGHT NURSES' HOURS.

"A. L." writes: The number of nurses who are always ready to complain only proves how many women enter the nursing profession who are totally unfitted for it. Surely the whole life of a nurse is, or should be, one of self-denial, and not only those hours in which she is on night duty. I have been a nurse for over six years, and am glad to say night duty has failed to demoralise me, as "A Lover of Justice" seems to fancy it can. Granted the twelve hours are full of work, one hardly expects to be able to "lose oneself in a book," as "A. M. M. B." suggests as possible when on duty in a large ward. Then as to sleeping quarters. In any good hospital these are always apart from the day nurses' rooms. They certainly were so in the well-known infirmary where I trained in the Midlands, and where I found that it is only the nurse who thinks it "fine" to be dainty, who complains of her food and the small ward kitchens where it is customary to eat it. What will such nurses do when they come to take private cases among all sorts and conditions of people? Perhaps then they will find that they have mistaken their vocation, and retire from a profession which at present they hardly seem to ennoble. I imagine that there always will be grumblers everywhere; but it seems to me that it is time for someone on the other side to say something in favour of the noblest profession for a true woman.

### NURSING IN THE SICK-WARDS OF CAMBERWELL WORKHOUSE.

"Another Nurse" writes: In looking over your valuable paper, dated February 17th, I was deeply interested in reading statements referring to Constance Road Workhouse. The cases mentioned I know to be correct, and I should further like to state that the day nurse has from 45 to 54 patients in each ward, for whom she alone is responsible. Sometimes her charge extends to two wards, which really means that she is responsible for 99 patients at one time from 1 P.M. to 7 P.M. or 10.30 P.M., as the case may be. The off-duty hours come to the nurse as an "oasis" in the desert, cheering drooping spirits; but the time is too short—from 1 P.M. to 11 P.M. once a week and each alternate Sunday from 1 P.M. to 10 P.M., and only the whole day once a year, except the annual leave. From 7 P.M. each evening the day nurse is free, except one Sunday in the month, when she is on duty from 7 A.M. to 10 P.M. In the mental wards the day nurse is often on late duty twice a week.

### CUBICLES AT FAVERSHAM.

"Minimus" writes: Seeing that the Faversham Guardians still lean towards the idea of cubicles, perhaps they might like to have the opinion of one who has suffered from the inconvenience and discomfort of sleeping in them. Should the nurses be on night duty, it is always more difficult to sleep in the daytime, and I for one have often been awakened by my neighbour in the next cubicle turning over in bed. Then, again, perhaps all do not wish to retire at the same time, and should they come an hour or more later ever so quietly the light sleeper is aroused, and may be a long time before going off again. If, too, one wants to get up early, all are disturbed. This, at least, is my experience. Surely nurses, as well as other people, want a few minutes quiet when off duty, but this is scarcely to be got if they are unfortunate enough to sleep in a cubicle. Much more might be said on the disadvantages of cubicles, but I think that these few things will suffice. I myself hope never to take an appointment again where there are any. However small the room, let us enjoy the comfort of it.

### A SAD CASE.

Miss Helen Todd, Matron of the Royal National Sanatorium, Bournemouth, writes: Will you kindly allow me, through the medium of your columns, to enlist the sympathies of those of your readers who have votes in the United Kingdom Benevolent Association on behalf of Nurse Catherine Flora Fanning. Her case is an especially sad one. She has been nursing for twenty-seven years, and is in her 57th year, and is almost entirely incapacitated through chronic gastritis and other ailments. She is a gentlewoman by birth, and for very many years helped largely to provide education for several nephews and nieces; she had, therefore, no chance of saving any money. She has neither relations in a position to help her nor any means of support beyond the exercise of her profession; for this she is now quite unfitted by physical infirmities.

### SKIMMED MILK.

"An Old Westminster" writes: As a London County Council lecturer and a trained nurse, I think it is a grave error for nurses to use skimmed milk at any time, which is apparently approved of in answers on cheap food in last week's Hospital. Would it not be better to have some fats than none? "Half a loaf is better than no bread." On the same principle, half a pint of fresh unskimmed milk diluted with water will be more nourishing than one pint of skimmed milk with no fats.

### Notes and Queries.

**REGULATIONS.**

**The Editor is always willing to answer in this column, without any fee, all reasonable questions, as soon as possible.**

**But the following rules must be carefully observed.**

1. **Every communication must be accompanied by the name and address of the writer.**
2. **The question must always bear upon nursing, directly or indirectly.**

**If an answer is required by letter a fee of half-a-crown must be enclosed with the note containing the inquiry.**

*Cleaning Sponges.*

(171) I should be glad to have some information with regard to the effective cleansing of marine sponges after operations. I should like to know the best possible way of disinfecting them.—*An Edmonton Nurse.*

First wash thoroughly in cold water, then in hot water about 110° F. Then soak for an hour in washing soda and water (warm), a handful of soda to a pint and a half of water. Rinse in running water to remove the soda, and then leave in 1 in 20 carbolic acid for one week or longer. This is not sufficient if the sponges have previously been used for a septic case.

*Catgut and Silk.*

(172) Can you give me various methods of sterilizing catgut and silk (for abdominal operations). Does very fine silk or catgut take the same time to sterilize as coarse, and which method is considered the most successful.—*Sister A. H.*

Silk should be boiled for half an hour in a 1 per cent. solution of soda. The simplest way of preparing catgut is to place it in a stopped bottle containing an aqueous solution of iodine and potassium iodide—1 per cent. of each. The catgut is ready for use at the end of eight days.

*Comparisons as to different Hospitals.*

(173) Will you kindly tell me which would be considered the better training—that given at Chichester General Infirmary, or that given at Chesterfield and North Derbyshire Hospital; also whether the district work that you would receive at the former during the three years would be an advantage or disadvantage?—*Anxious.*

We cannot express an opinion on the respective value of training in two similar institutions. Any knowledge gained with regard to district work is likely to be of value.

*Training in Massage.*

(174) Kindly tell me which is the best place in London to obtain massage training. I am anxious to get a first-class certificate. I am a trained nurse. Would Mrs. Creighton Hale's instruction rank higher than that at the National Hospital?—*Nurse D.*

It is a matter of individual preference. If you qualify by private instruction you must get the certificate of the Society of Masseuses. Write to the Secretary of the Incorporated Society of Masseuses, 12 Buckingham Street, Strand, W.C.

(175) Would you kindly tell me what text-books to procure and study prior to taking lessons with a view of becoming a certificated masseuse?—*C. I. E. W.*

"The Art of Massage," by Mrs. Creighton Hale, price 6s. You can procure it from the Scientific Press, 28 and 29 Southampton Street, Strand.

*Nursing Home in Cairo.*

(176) Kindly inform me in your queries column all you know concerning "The Hutchinson Nursing Home" at Cairo; also what kind of clothing is advisable for a trained nurse to take out with her.—*A. D.*

We cannot discuss private institutions in these columns. Write to the matron for full particulars. All nursing arrangements, however, are probably settled for this season. The clothing needed is chiefly light woollen. The mornings and evenings, especially the former, are often cold in Egypt, whilst the day is hot, so that care as to wraps and underclothing is necessary.

**Handbooks for Nurses.**

| | Post Free. |
|---|---|
| "A Handbook for Nurses." (Dr. J. K. Watson.) ... | 5s. 4d. |
| "Nurses' Pronouncing Dictionary of Medical Terms" | 2s. 0d |
| "Art of Massage." (Creighton Hale.) ... ... ... | 6s. 0d. |
| "Surgical Bandaging and Dressings." (Johnson Smith.) ... ... ... ... ... ... ... ... | 2s. 0d. |
| "Hints on Tropical Fevers." (Sister Pollard.) ... | 1s. 8d. |

Of all booksellers or of The Scientific Press, Limited, 28 & 29 Southampton Street, Strand, London, W.C.

### For Reading to the Sick.

EVENSONG.

I come to Thee to-night,
In my lone closet where no eye can see,
And dare to crave communion high with Thee,
Father of love and light!

Thou gavest the calm repose
That rests on all—the air, the birds, the flowers,
The human spirit in its weary hours,
Now at the bright day's close.

Not for myself alone
Would I these blessings of Thy love implore;
But for each penitent the wide world o'er,
Whom Thou hast called Thine own.

And for Thy heart's best friends
Whose steadfast kindness o'er my painful years,
Has watched to sooth affliction's grief and tears,
My warmest prayer ascends.

And now, O Father, take
The heart I cast with humble faith on Thee,
And cleanse its depths from each impurity,
For my Redeemer's sake!

*Anon.*

Peace is the beginning and end of prayer; its condition and its reward. Resign yourselves that ye may pray, and God will guard your thoughts, and hold them to Himself.

Distraction will come through weakness, ill-health, fatigue; only pray, guard, strive against it; humble yourselves under it, and for the past negligences, of which it is mostly the sad fruit; rely less upon yourselves, cast yourselves more upon God, hang more wholly upon Him, and long the more for that blessed time when the redeemed of the Lord shall serve Him day and night without distraction.

Prayer, especially, has its blessings, not only in common with other duties, in that it brings us into (if I may so speak) a community with God, that it is an acting according to His Will, that it opens our souls to receive more strength, but that it is direct speaking to Him. In it the soul is directly brought, as it were, face to face towards God, and He alloweth His Face to shine upon us, and us to turn towards Him, and to speak as a man speaketh to a father and a friend.

Each wish to pray is a breath from Heaven, to strengthen and refresh us; each act of faith, done to amend our prayers, is wrought in us by Him, and draws us to Him, and His gracious look on us. Each amends our prayers, and our amended prayers are His gifts to amend our lives, and so we may go on, in slow, it may be, through our manifold infirmities, yet ceaseless progress, until we find in His gracious and merciful Presence the end of our prayers, our faith, our hopes, our lives.

Strive we to pray, as we should serve God, with all our hearts and all our mind, and He will graciously help our infirmities, and accept our longings, and hear, we may even hope, what we longed to pray for, but failed.

